# Transcriptomic Profiling of Fe-Responsive lncRNAs and Their Regulatory Mechanism in Rice

**DOI:** 10.3390/genes12040567

**Published:** 2021-04-14

**Authors:** Shoudong Wang, Shuo Sun, Runze Guo, Wenying Liao, Huixia Shou

**Affiliations:** 1 State Key Laboratory of Plant Physiology and Biochemistry, College of Life Sciences, Zhejiang University, Hangzhou 310058, China; wangshoudong@iga.ac.cn (S.W.); sunshuo2019@zju.edu.cn (S.S.); grz@zju.edu.cn (R.G.); Liaowenying@zju.edu.cn (W.L.); 2 Key Laboratory of Soybean Molecular Design Breeding, Northeast Institute of Geography and Agroecology, Chinese Academy of Sciences, Changchun 130102, China; 3 The Provincial International Science and Technology Cooperation Base on Engineering Biology, International Campus of Zhejiang University, Haining 314400, China; 4 Hainan Institute, Zhejiang University, Sanya 572025, China

**Keywords:** lncRNAs, transcriptome, iron deficiency, rice, strand-specific RNA sequencing

## Abstract

Iron (Fe) deficiency directly affects crop growth and development, ultimately resulting in reduced crop yield and quality. Recently, long non-coding RNAs (lncRNAs) have been demonstrated to play critical regulatory roles in a multitude of pathways across numerous species. However, systematic screening of lncRNAs responding to Fe deficiency and their regulatory mechanism in plants has not been reported. In this work, 171 differently expressed lncRNAs (DE-lncRNAs) were identified based on analysis of strand-specific RNA-seq data from rice shoots and roots under Fe-deficient conditions. We also found several lncRNAs, which could generate miRNAs or act as endogenous target mimics to regulate expression of Fe-related genes. Analysis of interaction networks and gene ontology enrichment revealed that a number of DE-lncRNAs were associated with iron transport and photosynthesis, indicating a possible role of lncRNAs in regulation of Fe homeostasis. Moreover, we identified 76 potential lncRNA targets of OsbHLH156, a key regulator for transcriptional response to Fe deficiency. This study provides insight into the potential functions and regulatory mechanism of Fe-responsive lncRNAs and would be an initial and reference for any further studies regarding lncRNAs involved in Fe deficiency in plants.

## 1. Introduction

Iron (Fe) is an essential micronutrient for plants, but is often limited due to low availability in the soil [1]. To overcome Fe deficiency, plants have evolved two strategies to optimize Fe acquisition and uptake, i.e., the reduction strategy (Strategy I) for non-gramineous plants and the chelation strategy (Strategy II) for gramineous plants [2,3]. Rice, which is adapted to growing in the paddy field where the reduced form of Fe is available, utilizes both strategy I and strategy II Fe-uptake systems [4,5,6]. A large number of genes are known for their involvement in Fe uptake and homeostasis, including those that encode transcription factors for regulating expression of downstream Fe-responsive genes, enzymes for synthesis of phytosiderophores (MAs), and transporters of MA-Fe (III) or Fe (II) in rice [3,4,7,8,9,10,11,12,13,14]. To date, most of the regulators in the maintenance of plant iron homeostasis are coding genes, whether long non-coding RNAs (lncRNAs) play roles in Fe-deficiency response is still unknown.

LncRNAs are transcripts of more than 200 nucleotides in length but without coding potential that have recently gained widespread attention [15]. LncRNAs are classified as sense, antisense, intronic, and intergenic according to their position in relation to neighboring coding genes [16,17]. Conventional sequencing for mRNA is non-strand-specific, which is limited to identified cis-natural antisense lncRNAs as the antisense strand overlapping with a transcript on the opposite sense strand are unable to be distinguished. Strand-specific RNA-seq (ssRNA) is a powerful tool to definite the transcripts coming from which DNA strand, and be able to resolve overlapping transcripts and improve the accuracy of annotation [18].

Recent studies have shown that lncRNAs play important roles in numerous crucial biological processes across many species by regulating the expression of mRNAs at epigenetic, transcriptional, post-transcriptional, translational, and post-translational levels [15,19,20]. In plants, lncRNAs were reported to be involved in development and stress responses [21,22]. For instance, the lncRNAs *COLDAIR* (cold-assisted intronic noncoding RNA) and *COOLAIR* (cold-induced long antisense intragenic RNA) are both located in the *FLC* gene, which regulates flowering time. *COLDAIR* and *COOLAIR* regulate expression of *FLC* at the epigenetic level by interacting with an evolutionarily conserved repressive complex PRC2 (Polycomb Repressive Complex 2) [23,24,25]. Another lncRNA, long-day-specific male-fertility–associated RNA (*LDMAR*), regulates photoperiod-sensitive male sterility (PSMS) in rice [26]. A number of lncRNAs have been reported to regulate phosphate homeostasis. *IPS1* (induced by phosphate starvation 1) reduces phosphorus acquisition by inhibiting the activity of miR399, through the target mimicry mechanism [27]. The cis-natural antisense RNA (cis-*NATPHO1;2*), transcribed from *OsPHO1;2*, was found to be a translational enhancer of its sense gene (*OsPHO1;2*) [28]. In yeast, *prt* (*pho1*-repressing transcript), generated from the promoter region of the *pho1* gene, regulates expression of *pho1* responding to different phosphate levels [29]. These studies show a complicated network involving lncRNAs that regulate phosphate homeostasis.

Previous studies have shown a cross-talk between iron and phosphorus [30], the similar regulatory mechanism of lncRNA involved in Fe-deficiency response might also exist in plants. Therefore, genome-wide identification and characterization of lncRNAs responding to iron deficiency will enrich the regulatory network, and providing an opportunity for future computational and experimental studies to uncover the functions of lncRNAs under iron deficiency in rice. In this study, ssRNA library construction and RNA sequencing were performed to systematically identify and characterize any lncRNAs that respond to Fe deficiency.

## 2. Materials and Methods

### 2.1. Plant Growth Condition

*Oryza sativa* L. *cv. Nipponbare* (Nip) was used in this study. Seeds, from wild type (WT), *bhlh156* and *iro2* mutants, were germinated in the dark for 3 days, and then placed on a net floating on a solution with or without iron (1.43 mM NH_4_NO_3_, 1.0 mM CaCl_2_, 0.32 mM NaH_2_PO_4_, 1.64 mM MgSO_4_, 0.51 mM K_2_SO_4_, 0.13 μM CuSO_4_, 9.0 μM MnCl_2_, 0.08 μM (NH_4_)_6_Mo_7_O_24_, 0.02 μM H_3_BO_3_, 0.15 μM ZnSO_4_, 0.25 mM Na_2_SiO_3_, and 0 or 125 μM EDTA-Fe(II), pH 5.5–5.6). The nutrient solution was exchanged every 3 days. Rice plants were grown in a growth chamber at 30 °C during the day and 22 °C at night.

### 2.2. Strand-Specific RNA Library Construction and Sequencing

Shoots and roots were separately collected from seedlings (WT and *bhlh156* mutant) grown hydroponically for 10 days after germination with or without Fe, and frozen in liquid nitrogen immediately. Three biological replicates were used for each sample. Total RNA was extracted from these tissues using TRIzol (Invitrogen, Carlsbad, CA, USA) according to the manufacturer’s instructions. Strand-specific RNA (ssRNA) library construction and RNA sequencing were performed by the Beijing Genomics Institute in Shenzhen (BGI, Shenzhen, China). To construct the ssRNA library, the rRNA was removed with Ribo-Zero Gold rRNA Removal Kit (Epicentre, Madison, WI, USA) from the pooled RNA. The RNA was fragmented into 200–500 nts in length using fragmentation buffer. After synthesis of first-strand and second-strand cDNA, adapters were added to both sides of the short fragments. The second strand was degraded by uracil-N-glycosylase. The resulted single strand was PCR amplified and then sequenced by Illumina HiSeq PE151. The RNA-seq data is available in the NCBI (Accession number: PRJNA527175).

### 2.3. Identification and Characterization Pipeline of LncRNAs

The raw data obtained by Illumina sequencing was filtered into clean data by removing the adaptor, low quality reads and rRNA-containing reads with SOAPnuke and SOAP [31]. The dataset was aligned to the rice genome (Rice Genome Annotation Project) using the improved TopHat v 2.0 [32]. Cufflinks was used to reconstruct the transcripts. After filtering the background noise transcripts, the final expression data was produced [33]. Transcripts shorter than 200 bp were discarded. For the remaining sequences, the transcript coding potential values were predicted by the coding potential calculator (CPC) [34]. Each transcript with a CPC score < 0 were considered long non-coding RNAs. mRNA transcripts (CPC scores > 0) were also identified from the transcriptome in this work. Differentially expressed mRNAs or lncRNAs were identified using R package NOISeq 2.34 (https://bioconductor.org/packages/release/bioc/html/NOISeq.html; accessed on 13 April 2021).

### 2.4. Validation of Several LncRNAs Using RT-qPCR

Shoots and roots were sampled from seedlings of WT, *bhlh156* and *iro2* mutants, grown with or without iron for 10 days. Total RNA was isolated using TRIzol (Invitrogen) according to the manufacturer’s instructions. cDNA was synthesized from total RNA using a cDNA Synthesis Kit (TIANGEN, Beijing, China), and RT-qPCR was performed on a LightCycler480 machine (Roche, Mannheim, Germany) with SYBR Green Supermix (CWBIO, Beijing, China). ACTIN mRNA was used as the internal control for sample normalization. Means ± SD were calculated by three biological repeats. The RT-qPCR primers (synthesized by TSINGKE, Beijing, China) are shown in Table S1.

### 2.5. Prediction of the LncRNA-Derived miRNAs and Target Genes

For miRNA precursor analysis, the information of miRNA sequence and region in the genome were acquired from PmiREN (http://www.pmiren.com/; accessed on 13 April 2021) [35]. The miRNA was defined as lncRNA-derived miRNA if the pre-miRNA region in the genome was located in the lncRNA. For the target analysis, the online software psRNATarget (http://plantgrn.noble.org/psRNATarget/; accessed on 13 April 2021) was used to predict target genes of miRNAs with a maximum expectation of 2.0 [36]. Less than two mismatches and G/U pairs were allowed within the mRNA and miRNA pairing regions.

### 2.6. Prediction and Annotation of DE-LncRNA Targets

The potential target genes of differentially expressed lncRNAs (DE-lncRNAs) were predicted based on the two interaction modes between lncRNAs and mRNAs. For cis-target analysis, we searched coding genes located in 10 kb upstream or downstream of DE-lncRNAs. For the trans-target analysis, the interaction mode of DE-lncRNA and DE-mRNA was performed due to the complementary pairing of bases. The LncTar [37] tool was used for target gene prediction of lncRNAs. The free energy and standard free energy of paired sites were calculated, and the target genes with standard free energy threshold <−0.1 were considered as trans-target genes of lncRNAs, while <−0.2 for cis-target genes. The online software agriGO (http://systemsbiology.cau.edu.cn/agriGOv2; accessed on 13 April 2021) was used to do the GO enrichment, and only those biological process terms with *p* < 0.001 were considered as significantly enriched GO terms. The iron deficiency response lncRNA-mRNA networks were built using Cytoscape [38], which only contain DE-lncRNAs and the trans-targets, which had been reported as important Fe regulators.

## 3. Results

### 3.1. Genome-Wide Iidentification of LncRNAs

**Figure 1:**
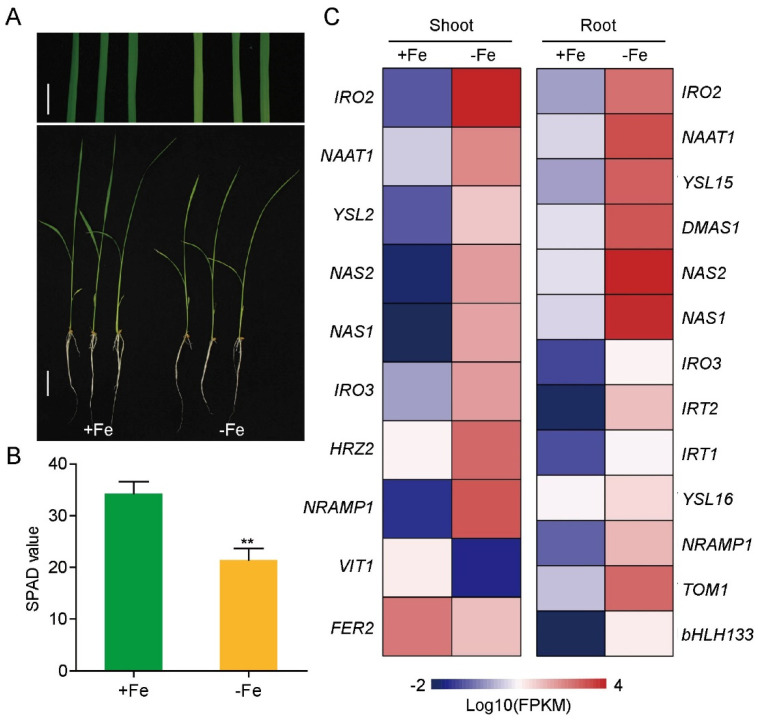
Physiological responses of rice seedlings to iron deficiency and expression pattern of Fe-related marker genes in RNA-seq. (**A**) Phenotypes of Nipponbare grown with 125 μM EDTA-Fe (II) (+Fe) or no Fe (-Fe) for 10 days (upper bar represents 1 cm, lower bar represents 3 cm). (**B**) Soil Plant Analysis Development (SPAD)values representing chlorophyll content of leaves. Data represent means ± SD, *n* = 4; ** *p* < 0.01, one-way ANOVA followed by the Tukey test. (**C**) Heatmap of Fe-related marker genes in rice shoot and root under iron deficiency.

To systematically identify and characterize lncRNAs in rice, ssRNA sequencing (ssRNA-seq) was performed on shoot and root samples from rice seedlings grown in Fe-sufficient and -deficient conditions. After 10 days of Fe-deficient growth, rice plants showed significant chlorosis and lower chlorophyll content in the young leaves (Figure 1A,B). The expression of typic Fe-deficiency responsive genes, such as the iron-related bHLH transcription factor 2 (*IRO2*), nicotianamine synthases 1 and 2 (*NAS1/2*), Fe(III)-DMA transporters (*YSL15/16*) and iron-regulated transporter 1 (*IRT1*), were significantly increased (Figure 1C), indicating that the rice seedlings were under iron deficiency condition at the sampling time.

**Figure 2:**
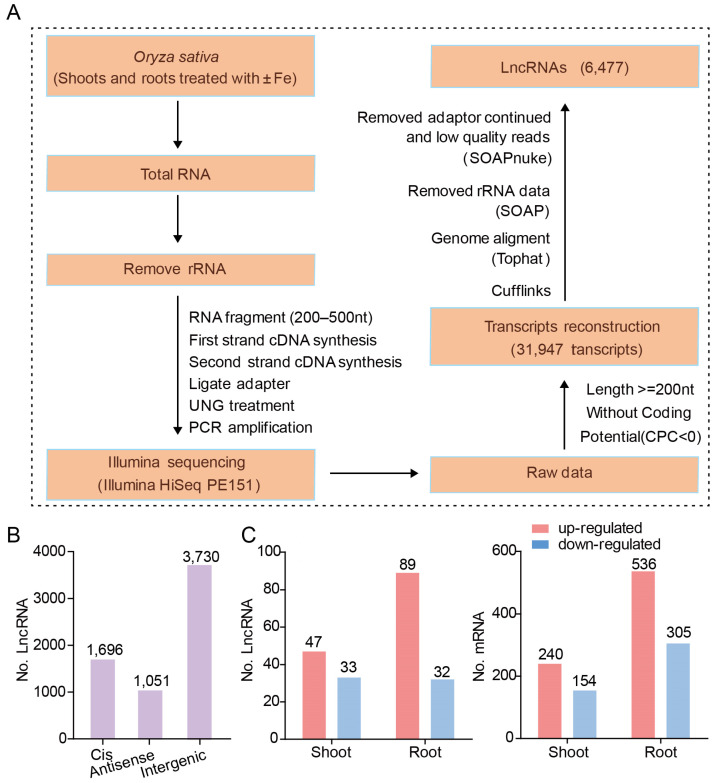
Identification and characterization of lncRNAs. (**A**) The pipeline for identification and characterization of known and novel lncRNAs responding to iron deficiency by ssRNA-seq (see methods). (**B**) Location of lncRNAs relative to the nearest protein-coding genes. LncRNAs were categorized according to the position relative to neighboring coding genes. The lncRNAs located at the antisense strand of coding transcripts were defined as antisense lncRNA. Other lncRNAs without overlap with coding transcripts were classified as intergenic lncRNAs, cis-lncRNAs were close (≤500 nt) and on the sense strand with the adjacent genes. (**C**) Total number of differentially expressed lncRNAs and mRNAs. The number of up-regulated (Log 2 (fold change) >1; fragments per kilobase of exon per million fragments mapped (FPKM) > 2; probability > 0.8) and down-regulated (Log 2 (fold change) < −1; FPKM > 2; probability > 0.8) lncRNAs and mRNAs in response to Fe deficiency in shoot and root.

The pipeline for lncRNA identification and characterization is shown in Figure 2A (see methods). Using this pipeline, approximately 700 million 150-bp pair-end reads were assembled into 31,947 transcripts using Cufflinks. Using this method, 25,470 mRNAs and 6477 lncRNAs were identified. Based on their relative position to protein-coding genes, lncRNAs can be classified into three types. Intergenic lncRNAs have no overlap with any protein-coding sequences, while sense lncRNAs and anti-sense lncRNAs overlap with one or more exons of another transcript on the same or opposite strand, respectively [21]. Among the 6477 lncRNAs identified in this work, 3730 (58%) were intergenic lncRNAs, 1696 (26%) were *cis*-lncRNAs, and 1051 (16%) were antisense lncRNAs (Figure 2B).

### 3.2. Fe-Deficiency Responsive LncRNAs and mRNAs in Rice Shoot and Root

To identify the lncRNAs and mRNAs that are differentially expressed in response to Fe deficiency, the normalized expression levels (in fragments per kilobase of exon per million fragments mapped, FPKM) of lncRNAs or mRNAs were compared between the Fe-deficient and Fe-sufficient treatments. In shoots, 80 DE-lncRNAs were identified. Among them, 47 lncRNAs were upregulated and 33 were downregulated (Figure 2C; Table S2A). In roots, 89 lncRNAs were upregulated and 32 were downregulated under Fe deficiency (Figure 2C; Table S2B). In addition, 394 and 841 mRNAs were differentially expressed in either roots or shoots due to Fe deficiency, respectively. In shoots, 240 mRNAs were upregulated and 154 were downregulated (Figure 2C; Table S2C), while in roots, 536 mRNAs were upregulated and 305 mRNAs were downregulated (Figure 2C; Table S2D).

**Figure 3:**
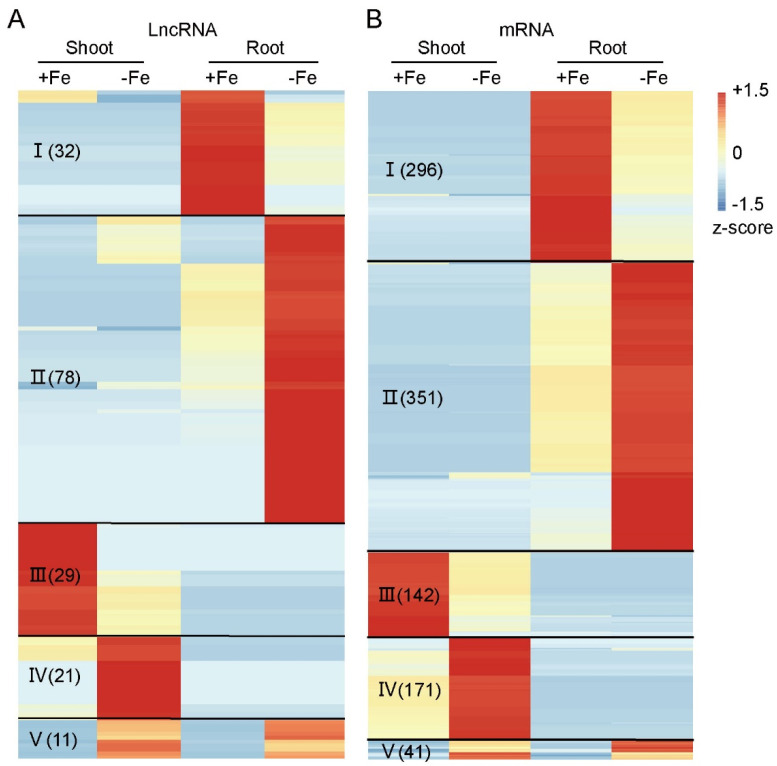
Heatmap of differentially expressed lncRNAs and mRNAs under ±Fe condition in both shoot and root. The lncRNAs (**A**) and mRNAs (**B**) identified as differentially expressed were used to make the heatmap. LncRNAs and mRNAs were divided the into five classes based on their expression patterns. Class I, highly expressed in root and downregulated under Fe deficiency. II, upregulated under Fe deficiency in root. III, highly expressed in shoot and down-regulated under Fe deficiency. IV, upregulated under Fe deficiency in shoot. V, upregulated both in shoot and root. The number of molecules in each class is listed in parentheses.

The DE-lncRNAs and -mRNAs were used to generate a heat map (Figure 3). Classes I and III contained lncRNA and mRNA transcripts that were expressed significantly higher in Fe-sufficient than in Fe-deficient conditions in either roots (Class I) or shoots (Class III), respectively. In contrast, transcripts in Classes II and IV had higher expression in roots or shoots under Fe-deficient conditions, respectively. Transcripts in Class V were more highly expressed in both shoots and roots under Fe-deficient conditions. Among the five groups, Class II, the transcripts induced under Fe-deficient roots, contained the largest number of both lncRNAs (Figure 3A) and mRNAs (Figure 3B). In total, 171 lncRNAs and 1001 mRNAs were differentially expressed under different Fe supply conditions (Figure 3; Table S3).

### 3.3. Verification of LncRNAs Responding to Fe Deficiency Using RT-qPCR 

**Figure 4:**
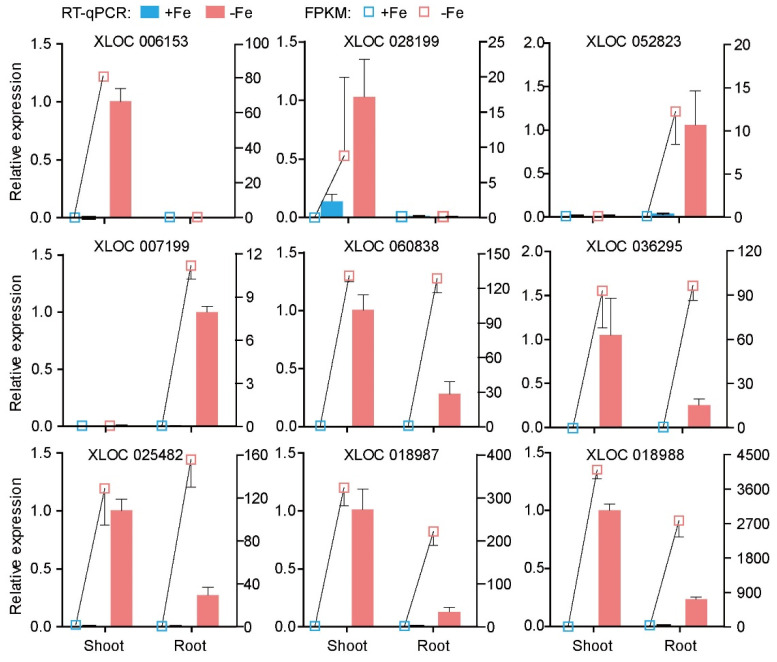
RT-qPCR validation of nine lncRNAs responding to Fe deficiency in RNA-seq transcriptome. Nine lncRNAs were chosen to perform RT-qPCR analysis. The relative expression level of the lncRNAs was presented on the left axis, the right showed FPKM value from RNA-seq transcriptome. Actin was used as a reference gene, means ± SD were determined from three biological repeats.

Quantitative real-time PCR (RT-qPCR) was performed to verify the accuracy of the RNA-seq data for the lncRNAs. Nine intergenic lncRNAs responding to Fe-deficiency were picked for the verification. The RT-qPCR results showed lncRNAs XLOC_006153 and XLOC_028199 from Class IV were induced in shoots but not detected in roots regardless of the Fe supply status. LncRNAs XLOC_052823 and XLOC_007199 from Class II were upregulated by Fe deficiency in the roots. The remaining five lncRNAs belonged to Class V, which were induced upon Fe deficiency in both shoots and roots (Figure 4). The consistent trend between the RNA-Seq and RT-qPCR result indicates the reliability of our transcriptomic profiling data.

### 3.4. Identification of LncRNAs as Potential miRNA Precursors and miRNA Target Mimics

**Figure 5:**
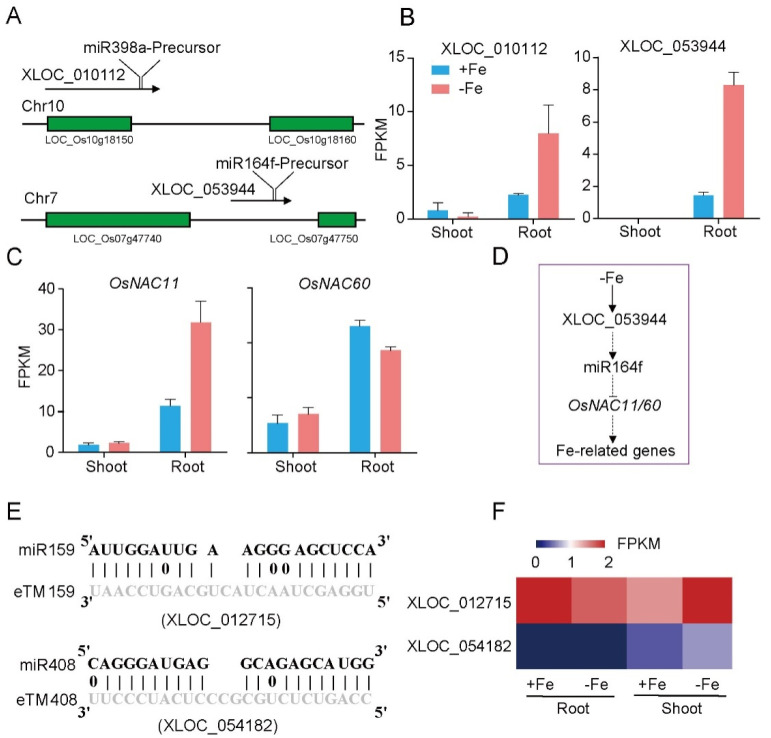
Predicted lncRNAs function as miRNA precursors and endogenous target mimics. (**A**) Schematic diagram of miRNA precursors and lncRNAs in rice genome. (**B**) The expression value of XLOC_010112 and XLOC_053944 in shoot and root. (**C**) The expression value of *OsNAC11* and *OsNAC60* in shoot and root. (**D**) Predicted working model for XLOC_053944. (**E**) Two endogenous target mimics (eTMs), osa-eTM159, and osa-eTM408 responding to iron deficiency in rice. The predicted base-pairing pattern between osa-miR159 and osa-miR408 and their eTMs. Base pairing between the miRNA and its lncRNA target mimic are shown, in which a vertical line means a Watson-Crick pair, two dots represent a G-U pair, and 0 means a mismatch. (**F**) The expression levels of osa-eTM159 and osa-eTM408 in the ssRNA-seq.

miRNAs regulate key aspects of development, cell signaling, and responses to various biotic and abiotic stresses via binding to specific complementary transcripts, including protein coding or non-coding sequences, resulting in the degradation or translational repression of the target. LncRNAs have been shown to function as precursors of miRNA in many studies [20,39]. By aligning miRNA precursors to the 171 DE-lncRNAs, 2 DE-lncRNAs, XLOC_010112, and XLOC_053944, were identified as the potential precursors of miR398a and miR164f, respectively (Figure 5A). XLOC_010112 is located in the region overlapped with an unexpressed coding gene (LOC_Os10g18150) (Figure 5A). Under Fe deficiency, XLOC_010112 was down-regulated in shoot and up-regulated in root (Figure 5B). XLOC_053944 was an intergenic lncRNA (Figure 5A), specifically expressed in root and significantly induced by Fe starvation (Figure 5B). Interestingly, miR398a and miR164f were both involved in regulation of Fe homeostasis in *Arabidopsis* [40,41]. The predicted target genes of miR398a and miR164f include LOC_Os06g23650, LOC_Os06g46270 (*OsNAC11*; *OsY37*; *OsMTN4*), LOC_Os12g41680 (*OsMTN3*; *OsNAC60*), and LOC_Os07g11360 (*RAL3*) (Table 1). Among them, *OsNAC11* was significantly induced, while *OsNAC60* was suppressed in root under Fe deficiency (Figure 5C). We speculated the work model of XLOC_053944: Under the Fe-deficiency condition, XLOC_053944 is induced in rice root and consequently generates miR164f. The increased amount of miR164f would reduce the transcript abundance of *ONAC11* and *OsNAC60*, which regulated the Fe-related genes as transcription factors (Figure 5D).

In addition to generating miRNAs, lncRNAs are also targets of miRNAs. In this case, lncRNAs function as target mimicry with the sequestered transcript known as an endogenous target mimic (eTM) to inhibit miRNA activity [27]. In order to further verify whether the target mimicry mechanism is involved in Fe regulation in rice, the potential interactions between the Fe-responsive lncRNAs and known Fe-related microRNAs were investigated. Two endogenous target mimics (eTMs), eTM159 and eTM408, were identified. The lncRNAs, XLOC_012715 (up-regulated in shoot and down-regulated in root under iron deficiency) and XLOC_054182 (only expressed in shoot, and slightly induced by Fe starvation), were predicted to bind the miR159 and miR408, respectively (Figure 5E,F). The potential target genes of miR159 or miR408 were listed in Table 1, including MYB transcription factors, *OsGAMYB*, *OsGAMYBL1*, and calmodulin-like protein *OsCML27*. The results demonstrated that target mimicry might be a part of the regulation of Fe homeostasis.

### 3.5. Interactions of DE-LncRNAs with mRNAs

**Figure 6:**
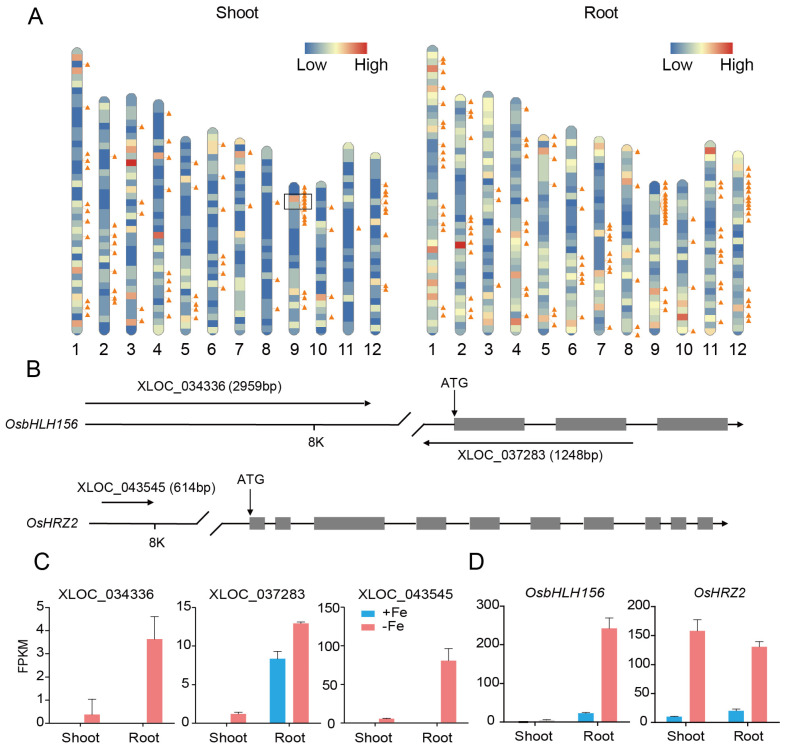
Predicted lncRNAs function as cis-regulators to regulate the related protein-coding genes. (**A**) Distribution of differentially expressed lncRNAs (DE-lncRNAs) and -mRNAs on the 12 rice chromosomes. The transcripts that were differentially expressed in shoots and roots were mapped to the chromosomes separately for clarity. Different colors represent different densities of mRNAs on the chromosome, with red color denoting a high density and blue denoting a low density. Each triangle represents one lncRNA identified in this study. (**B**) Schematic diagram of three DE-LncRNAs at genomic regions of *OsbHLH156* and *OsHRZ2*. (**C**,**D**) Expression value of three LncRNAs (**C**) and *OsbHLH156* and *OsHRZ2* (**D**).

Recent studies have shown that lncRNAs regulate the expression of genes via either cis- or trans-acting modes based on their genomic proximity to protein-coding genes [42]. The genomic locations of the DE-lncRNAs and DE-mRNAs were mapped to each chromosome of the rice genome. The results indicated that both DE-lncRNAs and DE-mRNAs were evenly distributed to each chromosome other than two regions in chromosome 9 and 12, which showed higher degree clustering of DE-lncRNAs, more interesting was that there were also many DE-mRNAs accumulated near the region in chromosome 9 (Figure 6A). For cis-target analysis, 12 DE-mRNAs spaced less than 10 kb away from 15 DE-lncRNAs in shoot (Table S4A) and 34 DE-mRNAs spaced less than 10 kb away from 37 DE-lncRNAs in root (Table S4B) were identified. These lncRNA-franking coding genes including bHLH transcription factors, E3 ubiquitin ligases, and tyrosine protein kinases. Interestingly, three lncRNAs were found to be located nearby *OsbHLH156* and hemerythrin motif-containing really interesting new gene (RING)- and zinc-finger protein 2 (*OsHRZ2*), which were two important regulators involved in Fe homeostasis in rice (Figure 6B) [14,43]. LncRNA XLOC_037283 and XLOC_034336 are located in *OsbHLH156* or nearby. XLOC_037283 is likely an antisense transcript (NAT) locating to the part of the promoter and coding region of *OsbHLH156* in opposing orientation, while XLOC_034336 is in 8 kb upstream ATG of *OsbHLH156*. Both lncRNAs showed a similar expression pattern with *OsbHLH156* under the Fe deplete condition (Figure 6C). XLOC_043545 is located in 8 kb upstream ATG of *OsHRZ2* and mainly expressed in rice root under iron deficiency, while *OsHRZ2* could be induced in both shoot and root (Figure 6D). The results demonstrated lncRNA might play a role in Fe signaling pathway as cis-regulators, and likely involved in transcriptional or post-transcriptional regulation of *OsbHLH156* and *OsHRZ2*.

**Figure 7:**
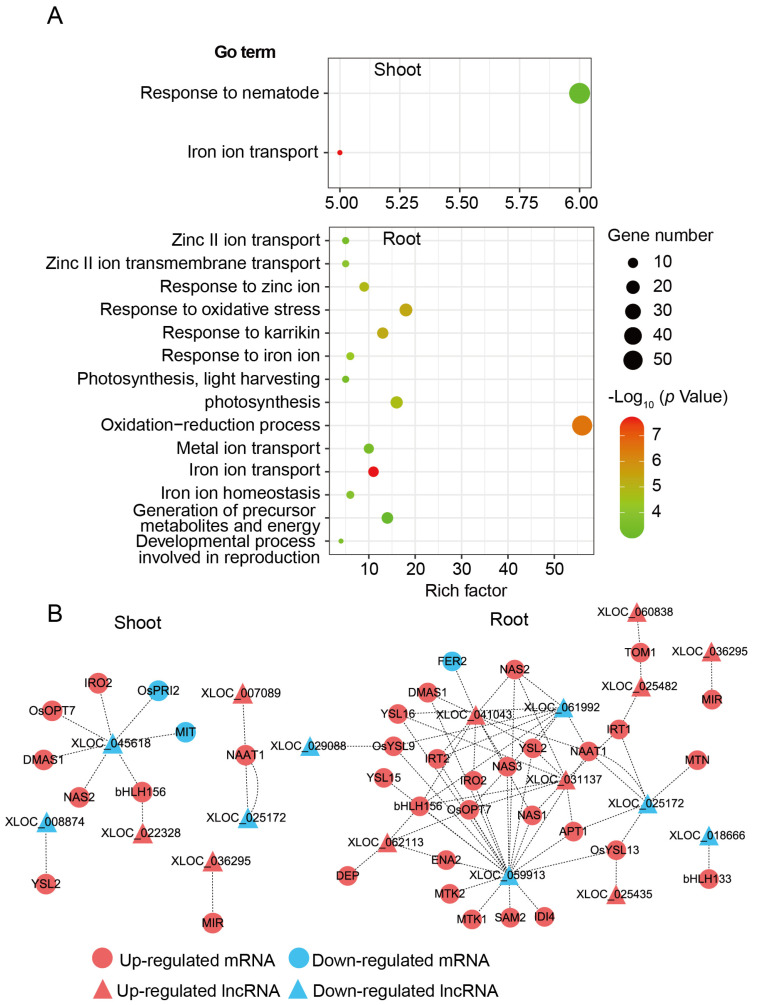
Interaction of DE-lncRNAs with DE-mRNAs. (**A**) Gene ontology (GO) enrichment of DE-mRNAs predicted to interact with DE-lncRNAs in shoot and root (*p* < 0.001). Only biological process terms were shown. (**B**) Interaction networks among DE-lncRNAs and Fe-related mRNAs based on complementary pairing of bases in shoot and root. Circles and triangles represent Fe-related mRNAs and DE-lncRNAs, respectively. Red and blue represent upregulated and downregulated, respectively. Dotted line shows the potential interaction between mRNA and lncRNA.

For trans-target analysis, 478 interaction modes of DE-lncRNA and DE-mRNA in shoot and 1516 in root were inferred according to the complementary pairing of bases (Table S5). Furthermore, GO enrichment analysis was performed to investigate the potential functions of the trans-target genes. As shown in Figure 7A, we found 14 GO terms that were significantly enriched in root but only 2 in shoot. Among them, “Response to iron ion”, “Metal ion transport”, “Iron ion transport”, and “Iron ion homeostasis” were all associated with response to Fe-deficient stress. Interaction modes among DE-lncRNAs and Fe-related genes were selected to perform the interaction networks, six DE-lncRNAs were found, which could interact with more than five Fe-related genes (Figure 7B). The results implied a complex regulation network between lncRNAs and mRNAs might contribute to Fe homeostasis regulation in rice.

### 3.6. DE-LncRNAs Regulated by Transcription Factors bHLH156 and IRO2 at the Transcriptional Level

**Figure 8:**
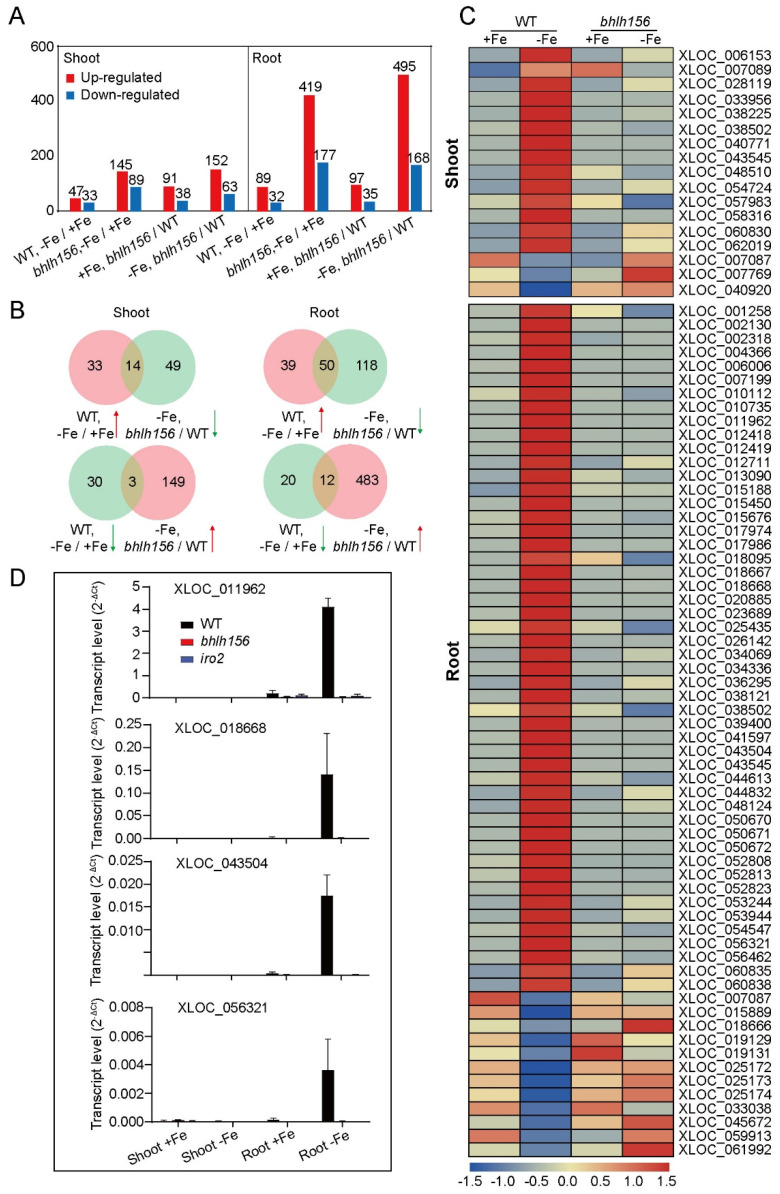
DE-LncRNAs regulated by bHLH156 and IRO2. (**A**) Number of DE-lncRNAs in shoots and roots of *bhlh156* mutant under Fe deficiency. The filtering rules is same with that in Figure 2C. (**B**) Venn diagram. (**C**) Heatmap constructed by DE-lncRNAs responded in an antagonistic manner in rice roots under Fe deficiency. The color scale indicates the *z*-score associated with the DE-lncRNAs. (**D**) Transcript abundance of DE-lncRNAs in shoot and root of WT, *bhlh156* and *iro2*. Expression was detected by RT-qPCR. Transcript levels were calculated relative to *OsActin*.

To test whether expression of DE-LncRNAs could be regulated by Fe-related transcription factors, a ssRNA-seq was performed on shoot and root samples from knock out mutant of bHLH156 grown in Fe-deficient and Fe-sufficient conditions, which act as a core transcription factor in regulating Fe homeostasis together with IRO2 [14]. The DE-lncRNAs in both *bhlh156* shoot (145 upregulated and 89 downregulated) and root (419 upregulated and 177 downregulated) were more than that in WT (Figure 8A, Table S7). Under Fe deficiency, 495 lncRNAs were up-regulated and 168 were down-regulated in *bhlh156* root when compared with WT (Figure 8A, Table S7). LncRNAs responded in an antagonistic manner in rice roots under Fe-deficiency condition were most likely regulated by bHLH156. As shown in Figure 8B,C, 14 Fe-deficiency-induced lncRNAs in shoots and 50 in roots of WT were suppressed in *bhlh156* mutant. In addition, 3 and 12 downregulated lncRNAs expressed in shoots and roots, respectively, of WT showed a significantly higher expression level in *bhlh156* (Table S8). To verify that these lncRNAs were truly regulated by bHLH156, four of them (XLOC_011962, XLOC_018668, XLOC_043504, and XLOC_056321) were chosen to perform RT-qPCR analysis in *bhlh156* mutant, also in *iro2* mutant that is a necessary interacting partner for bHLH156 to activate the downstream genes [14]. XLOC_011962, XLOC_018668, XLOC_043504, and XLOC_056321 were all specifically expressed in root and dramatically induced under Fe-deficiency in WT, but barely detected in *bhlh156* and *iro2* mutants (Figure 8D). The results demonstrated that a number of DE-LncRNAs could be regulated by bHLH156 and IRO2 at the transcriptional level.

## 4. Discussion

LncRNAs play important roles in a wide range of biological processes, including development, stress responses, and plant nutrition. In this work, lncRNAs that respond to Fe deficiency in rice roots and shoots were identified. Results generated in this study promote our understanding of how rice plants respond to Fe deficiency.

LncRNAs arise from intergenic, intronic, or coding regions in the sense and antisense directions with a lower expression level than mRNAs. Thus, identification of lncRNAs requires the use of an ssRNA-seq strategy. In this study, 6477 lncRNAs were identified and characterized (Figure 2A). Differentially expressed lncRNAs and mRNAs were identified by comparing the expression level between +Fe and –Fe conditions. The expression patterns divided them into five classes (Figure 3). Class II, which comprised the molecules up-regulated in the rice root, had the greatest number of transcripts. Among the differentially expressed lncRNAs and mRNAs, we found that the number of lncRNAs and mRNAs responding to Fe deficiency had a similar trend, with more lncRNAs and mRNAs upregulated under Fe deficiency in both shoots and roots. Moreover, a greater number of lncRNAs and mRNAs were detected in roots in the Fe-deficient condition. 

miRNAs are small RNAs that regulate target genes at both transcriptional and post-transcriptional levels, and generated by sequential cleavage of long precursor transcripts. Some lncRNAs could also act as primary transcripts of miRNAs [39]. In this study, two precursors of miRNAs generated from lncRNAs were identified, in which XLOC_053944 might produce miR164f to degrade *OsNACs* mRNA (Figure 5A–D). It has been shown that conserved miR164-targeted *NAC* acts as negative regulators of drought tolerance in rice [44]. In addition, the *NAC* member family has also been found to play an important role in Fe homeostasis [10]. Further investigation should be conducted to verify whether miR164-targeted *NAC* also participate in Fe regulation, which might be a cross talk between drought tolerance and Fe-deficiency response.

LncRNAs have been shown to regulate phosphate homeostasis in plants by a novel mechanism called target mimicry [27,45]. Two endogenous lncRNA target mimics (eTMs) were identified in rice, namely eTM159 and eTM408, which target two Fe-related miR159 and miR408 (Figure 5E) [41]. Therefore, a similar mechanism of *IPS1*-mi399 in regulating phosphate homeostasis via target mimicry also exists in Fe regulation. We speculated that when rice upon the iron deficiency condition, XLOC_012715 and XLOC_054182 targeted miR159 and miR408, and prevented them to degrade coding genes, including zinc finger protein, calmodulin-like protein 27, *OsGAMYB*, and *OsGAMYB* (Table 1). Most of target genes were not Fe-related genes except for *OsCML27* (LOC_Os03g21380), which was up-regulated in root, a possible reason is this target mimicry might only happen in specific cell types, the changes of expression level of target genes could not be detected well using whole shoot or root tissues.

LncRNAs have been shown to regulate expression of adjacent genes via recruiting regulatory complexes through RNA–protein interactions, or correlate with expression of neighboring genes acting as local regulators [46,47]. In order to study whether this mechanism is involved in Fe regulation, we compared DE-lncRNAs location to 10 kb upstream and downstream of 64 known Fe-related genes (Table S6), only three DE-lncRNAs were identified nearby *OsbHLH156* and *OsHRZ2* (Figure 6B), which are two important regulators involved in the Fe signaling pathway. *OsbHLH156* regulates Strategy II iron acquisition as a core transcription factor [14] and *OsHRZ2* is a putative iron-binding sensor that negatively regulates iron acquisition under Fe-sufficiency condition [43]. Both *OsbHLH156* and *OsHRZ2* are strongly induced by Fe deficiency, however, transcriptional and post-transcriptional regulation of these two genes is largely unknown. In this study, we found two DE-lncRNAs, XLOC_034336 and XLOC_043545, were located in the upstream of *OsbHLH156* and *OsHRZ2*, respectively. XLOC_037283 is a NAT and overlapped within genome of *OsbHLH156*, and showed a synchronous expression pattern with *OsbHLH156* (Figure 6B–D). A series of case studies have shown that NAT could either positively or negatively regulate expression of the cognate loci [48]. *Arabidopsis P5CDH* (Δ^1^-pyrroline-5-carboxylate dehydrogenase) and *SRO5,* an overlapping gene of unknown function in the antisense orientation, generate both 24-nt and 21-nt siRNAs to regulate *P5CDH* at the post-transcriptional level. *P5CDH-SRO5* gene pair defines a mode of siRNA function, which may be applied to other *cis*-antisense gene pairs [49]. *COOLAIR* is transcribed in antisense orientation in relation to *FLC*, have an early role in the epigenetic silencing of *FLC*, acting to silence *FLC* transcription transiently [23]. Moreover, NAT could also increase in mRNA stability by forming a duplex like *BACE1* locus in mammalian [50]. A similar regulation mechanism as mentioned above might also exist between XLOC_037283 and *OsbHLH156*, therefore, more precise and detailed spatiotemporal expression pattern of XLOC_037283 and *OsbHLH156* upon Fe deficiency, and detection of methylation level at the *OsbHLH156* locus should be conducted to verify the conjecture. These DE-lncRNAs are very necessary to conduct further study, because which might regulate expression of *OsbHLH156* and *OsHRZ2* under iron deficient condition, this mechanism will strengthen the Fe regulation network in rice. 

The DE-LncRNAs described above mainly function as upstream regulators of Fe-related genes, however, transcriptional regulation of these Fe-deficiency responsive lncRNAs was still unknown. To study whether DE-lncRNAs could also be regulated by Fe-related genes, a same ssRNA-seq was also performed using the *bhlh156* mutant. In this study, a total of 76 DE-lncRNAs (in shoot or root, or both) were identified and whose expression might be activated or inhibited by transcription factor bHLH156 (Figure 8C, Table S8), which has been proved to be required for induction of nearly all Strategy II iron acquisition genes in rice [14]. RT-PCR was performed to verify the accuracy of expression of these DE-lncRNAs using *bhlh156* and *iro2* mutants. The results indicate that bHLH156-IRO2 complex might also regulate Fe homeostasis via activating downstream lncRNAs. 

## 5. Conclusions

In conclusion, the study shows that there is a group of lncRNAs regulating the expression of Fe-related genes. The regulatory mechanisms involve biogenesis of miRNAs, interacting with miRNA function as endogenous target mimics, or cis/trans-acting modes. On the other hand, the expression of a number of lncRNAs is under the control of the core Fe-responsive transcription factors *OsbHLH156* and *OsIRO2*. These lncRNAs might form a feedback loop to modulate the Fe-deficiency response. We believe our study would be an initial and reference for understanding the function of the lncRNAs in regulating iron homeostasis in rice.

## Figures and Tables

**Table 1:** Predicted target genes of miRNA164, -159, and -408. Inhibition: cleavage.

miRNA	Target	Description	miRNA and Target Aligned_Fragment
miR164f	LOC_Os06g23650	No apical meristem protein	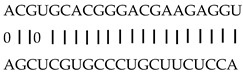
miR164f	LOC_Os06g46270	*OsNAC11*; *OsY37*; *OsMTN4*	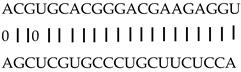
miR164f	LOC_Os12g41680	*OsMTN3*, *OsNAC60*	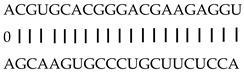
miR159a	LOC_Os10g05230	Zinc finger, C3HC4 type domain containing protein	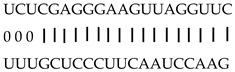
miR159a/b	LOC_Os01g59660	*OsGAMYB*	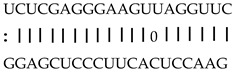
miR159a/b/c/d/e	LOC_Os01g12700	MYB transcription factor	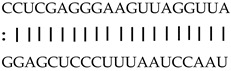
miR159a/b/c/d/e	LOC_Os05g41166	MYB transcription factor	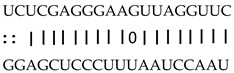
miR159a/b/c/d/e	LOC_Os09g36650	Expressed protein	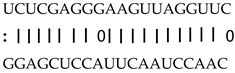
miR159b	LOC_Os03g21380	*OsCML27*, calmodulin-like protein	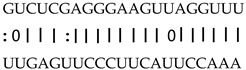
miR159c/d/e	LOC_Os06g40330	*OsGAMYBL1*	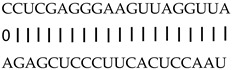
miR408	LOC_Os07g43540	*ORC6*	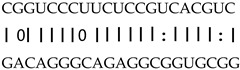

## Data Availability

The RNA-seq data is available in the NCBI (Accession number: PRJNA527175).

## Supplementary Materials

The following are available online at https://www.mdpi.com/article/10.3390/genes12040567/s1, Table S1: Primers used in this study for validation of lncRNAs. Table S2: LncRNAs and mRNA significantly up- or downregulated by Fe deficiency in shoot and root. Table S3: All differently expressed lncRNAs and mRNAs. Table S4: Cis-targets of DE-lncRNAs in shoot and root. Table S5: Trans-targets of DE-lncRNAs in shoot and root. Table S6: List of Fe-related genes. Table S7: LncRNAs significantly up- or down-regulated by Fe deficiency. Table S8: List of DE-lncRNAs in shoot used for constructing heatmap.
